# Adrenal fatigue does not exist: a systematic review

**DOI:** 10.1186/s12902-016-0128-4

**Published:** 2016-08-24

**Authors:** Flavio A. Cadegiani, Claudio E. Kater

**Affiliations:** From the Adrenal and Hypertension Unit, Division of Endocrinology and Metabolism, Department of Medicine, Escola Paulista de Medicina, Universidade Federal de São Paulo (EPM/UNIFESP), R. Pedro de Toledo 781–13th floor, 04039-032 São Paulo, SP Brazil

**Keywords:** Adrenal depletion, Adrenal fatigue, Cortisol, Adrenal insufficiency, Burnout, Fatigue

## Abstract

**Background:**

The term “adrenal fatigue” (“AF”) has been used by some doctors, healthcare providers, and the general media to describe an alleged condition caused by chronic exposure to stressful situations. Despite this, “AF” has not been recognized by any Endocrinology society, who claim there is no hard evidence for the existence. The aim of this systematic review is to verify whether there is substantiation for “AF”.

**Methods:**

A systematic search was performed at PUBMED, MEDLINE (Ebsco) and Cochrane databases, from the beginning of the data until April 22nd, 2016. Searched key words were: “adrenal” + “fatigue”, “adrenal” + “burnout”, “adrenal” + “exhaustion”, “hypoadrenia”, “burnout” + “cortisol”, “fatigue” + “cortisol”, “clinical” + “burnout”, “cortisol” + “vitalility”, “adrenal” + “vitality”, and “cortisol” + “exhaustion”. Eligibility criteria were: (1) articles written in English, (2) cortisol profile and fatigue or energy status as the primary outcome, (3) performed tests for evaluating the adrenal axis, (4) absence of influence of corticosteroid therapy, and (5) absence of confounding diseases. Type of questionnaire to distinct fatigued subjects, population studied, tests performed of selected studies were analyzed.

**Results:**

From 3,470 articles found, 58 studies fulfilled the criteria: 33 were carried in healthy individuals, and 25 in symptomatic patients. The most assessed exams were “Direct Awakening Cortisol” (*n =* 29), “Cortisol Awakening Response” (*n =* 27) and “Salivary Cortisol Rhythm” (*n =* 26).

**Discussion:**

We found an almost systematic finding of conflicting results derived from most of the studies methods utilized, regardless of the validation and the quality of performed tests. Some limitations of the review include: (1) heterogeneity of the study design; (2) the descriptive nature of most studies; (3) the poor quality assessment of fatigue; (4) the use of an unsubstantiated methodology in terms of cortisol assessment (not endorsed by endocrinologists); (5) false premises leading to an incorrect sequence of research direction; and, (6) inappropriate/invalid conclusions regarding causality and association between different information.

**Conclusion:**

This systematic review proves that there is no substantiation that “adrenal fatigue” is an actual medical condition. Therefore, adrenal fatigue is still a myth.

## Background

The term “adrenal fatigue” (“AF”) has been used by some doctors, healthcare providers, and the general media to describe an alleged condition caused by chronic exposure to stressful situations. According to this theory, chronic stress could potentially lead to “overuse” of the adrenal glands, eventually resulting in their functional failure. In a recent search on Google (April 22, 2016), “adrenal fatigue” provided 640,000 results, and the association of the two words exhibited 1,540,000 findings. Despite this, “adrenal fatigue” has not been recognized by any endocrinology societies to date, who claim there is no evidence for the existence of this syndrome [1].

Conversely, some medical societies, although unrecognized by American Board of Medical Specialties and Association of American Medical Colleges [2, 3], claim that adrenal fatigue is a real and underdiagnosed disease [4, 5]. According to these societies, to screen for “AF” in patients, a questionnaire developed by Dr. Wilson, who is reportedly the first person to describe this supposed syndrome, is recommended to be used [6]. In addition, patients suspected of “AF” are now being tested for serum basal cortisol levels and salivary cortisol rhythm. Those who present impaired results from these tests are then treated with corticosteroids, regardless of the etiology. As a result, corticosteroids (mainly hydrocortisone) are probably being prescribed to a large number of patients, as at least 24,000 health providers [7] are instructed by one medical society (The American Academy of Anti-Aging Medicine – A4M) to prescribe corticosteroids in these cases.

Arguments for corticosteroid use as a treatment for “claimed AF” include: [1] the immediate and significant improvement seen in patients who are prescribed corticosteroid, and [2] the long and extensive clinical symptomatology of this alleged disease, which shows a slow depletion before clinical and severe hypocortisolism ensues [4–6]. Moreover, others claim that endocrinologists use much too strict diagnostic criteria before prescribing corticosteroids, and thus, many sufferers would not be receiving adequate treatment [4, 6]. However, there are logical counterarguments to routine corticosteroid use in these patients. First, corticosteroids promote a sense of wellbeing (usually temporary), regardless of the patient’s condition. Second, even at low and physiological doses, corticosteroids increase the risk for several disorders, such as psychiatric disorders [8–11], osteoporosis [12], myopathy [13], glaucoma [14], metabolic disorders [14, 15], sleep disturbances [16] and cardiovascular diseases [17, 18].

Therefore, is “adrenal fatigue” an actual disorder? Is fatigue related to depleted adrenal function? Does fatigued healthy subjects present relative adrenal failure? Is adrenal involved in the pathophysiology of fatigue in diseases? Which tests were performed in order to establish markers or triggers? The aim of this systematic review was to determine the correlation between adrenal status and fatigue states, including the recently described “burnout” or “burnout syndrome”, and other fatigue-related diseases. The primary objective was to evaluate the methodology for fatigue status assessment, including cortisol tests, and to examine the results of studies involving cortisol and fatigue correlation.

## Methods

### Search strategies

The PRISMA protocol for systematic reviews was utilized for this study design. A systematic search was conducted through the electronic PUBMED, MEDLINE (Ebsco), and COCHRANE databases, from the beginning of the data until April 22, 2016. The search strategy included the following keywords: (1) “adrenal + fatigue”; (2) “adrenal + burnout”; (3) “adrenal + exhaustion”; (4) “adrenal” + “fatigue”; (5) “hypoadrenia”; (6) “cortisol” + “fatigue”; (7) “cortisol” + “burnout”; (8) “clinical” + “burnout”; (9) “cortisol” + “vitality”; (10) “adrenal” + “vitality”; and (11) “cortisol” + “exhaustion”, where “a + b” means “a” and “b” together in the exact expression, and “a” +”b” means that both words needed to be contained in the article, but not necessarily together. Although the terms “adrenal + fatigue” and “adrenal” + “fatigue” were searched, as articles found using the first criteria were also found using the second criteria, further analysis were performed for the exact expression that matched with the disease. We also analyzed articles mentioned within identified studies whenever the alleged disorder, or a similar situation, were described (such as cortisol profile and exhaustion or fatigued patients).

### Data extraction

All studies were evaluated by the two reviewers (F.A.C. and C.E.K.) after removal of duplicate articles, according to: (1) authorship, (2) journal, (3) publication date, (4) studied population, (5) definition of “fatigue”, “exhaustion”, and “burnout”, (6) study design and methods, (7) analysis methods to assess adrenal axis, (8) results, (9) conclusions, and (10) study variables and bias.

### Inclusion and exclusion criteria

Inclusion criteria were: (1) whole article written in English, (2) cortisol profile and fatigue or energy status as the primary outcome, (3) specific tests performed for evaluating the adrenal axis, (4) absence of corticosteroid therapy, (5) absence of confounding diseases that would lead to an impaired cortisol status caused by the disorder itself (such as depression, alcoholism, and morbid obesity). Studies with a merely description about adrenal axis impairment with no tests performed were excluded.

### Quality assessment

The abstract of each of identified study was analyzed by one of the authors (F.A.C.), and was excluded if it did not meet the eligibility criteria. The studies that fulfilled the inclusion criteria were entirely evaluated regarding the rationale, method design, primary outcome, assessment of fatigue, statistical analysis, results, discussion, and conclusions, in order to improve data quality. Those studies that presented any bias in the methodology, results, or interpretation of the exposed data, which could be reflected in the analysis of the study as a whole, were also excluded.

### Statistical analysis

Each of the studied populations, each of the questionnaires and each of the tests performed were quantified, whereas tests results were analyzed in terms of percentage of type of responses for each of the tests performed. Results were analyzed in general and according to the underlying disease.

## Results

### Study selection

**Fig. 1:**
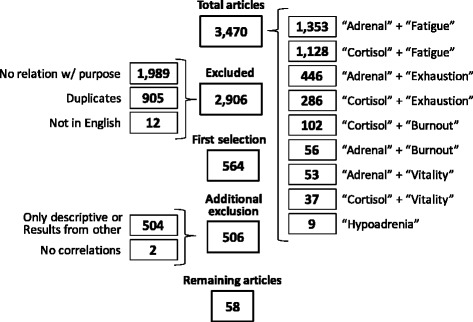
Study selection

In total, 3,470 articles were identified. A summary of the study selection is shown in Fig. 1. The search for “adrenal” + “burnout” yielded 56 studies; “adrenal” + “exhaustion” yielded 446 articles; “adrenal” + “fatigue” yielded 1,353 articles; “fatigue” + “cortisol” yielded 1,128 articles; “cortisol” + “burnout” yielded 102 articles; “cortisol” + “vitality” yielded 37 articles; “adrenal” + “vitality” yielded 53 articles; “hypoadrenia” yielded 9 articles articles (“hypoadrenocorticism” yielded 1,302 articles but is used to refer to hypocortisolism in animals, and therefore, was not included here); and “cortisol” + “exhaustion” yielded 286 articles. Twelve studies were excluded because they were written in languages other than English, 1,989 were excluded because there was no relation with the purpose of the systematic review, whereas 905 articles of interest were duplicates. Of the 564 remaining studies, 504 had only descriptive characteristics or contained results already presented in another study (in which tests were performed), and therefore were excluded. Two studies were excluded because despite of the correlation between cortisol profile and burnout or multiple sclerosis, they did not perform correlation between fatigue and cortisol, but other aspects, as depression and pain [19, 20]. For the systematic review, we analyzed all the included and not excluded studies, which represent a total of 58 articles (1.67 % of the original search) (Table 1).

**Table 1:** Summary of selected studies

First Author & Reference	Year of Publication	Number of Patients	Population	Questionnaire	Tests	Results	Comments
McLennan [21]	2016	257	H/B	Maslach	HCC	Nl	
Schmidt [69] (part 1)	2016	265	Breast Cancer	FAQ	DAC, CAR, SCR, NSC	↑NSC, ↑AUC, Nl CAR, Nl DAC, ↓SCR	Pts. mostly on chemotherapy (ChTx); schemes not specified. No controls, only correlation between fatigue levels and tests. ChTx related to worse fatigue. Initial tests performed during, w/ or w/o ChTx.
Schmidt [69] (part 2)	2016	265	Breast Cancer	FAQ	DAC, CAR, SCR, NSC	↑AUC, ↑CAR, ↑NSC, ↑SCR, Nl DAC	Second test of the study, performed after 14 weeks of procedures.
Sjors [25]	2015	220	H/B	SMBQ	DAC, CAR	↓CAR, ↑DAC	Results normalized after adjustment of anti-depressive use; SCR results not provided
Oosterholt [28]	2015	91	H/B	Maslach	DAC, CAR, SCR, AUC, CAR 60 min	Nl SCR, ↓CAR, Nl AUC, Nl CAR 60 min, ↓DAC	Control of variations did not change results
De Vente [22]	2015	95	H/B	Acute Psychosocial Stressor diagnosis	MSC, MST	Nl MSC, Nl MST	MST: Nl (women), reduced (men); MSC: Nl (men), reduced (women). Mental arithmetic and public speech stressors also performed
Lennartsson [24]	2015	56	H/B	DCSRD; exclusion w/ SEQ	DHEA-S; ACTH; ACTH and DHEA-S post-TSST (TSST stress test performed)	↓DHEA-S, ↓post-TSST DHEA-S, Nl ACTH, Nl post-TSST ACTH	
Tao [23]	2015	171	H/B	Maslach	MSC, ACTH	↑ACTH, ↑MSC	
Jonsson [29]	2015	51	H/B	SMBQ	MSC, MST	Nl MSC, ↓MST	
Lennartsson [27]	2015	56	H/B	DCSRD and SMBQ	MSC, MST, ACTH, ACTH post-TSST (TSST stress test performed)	Nl MSC, Nl MST, Nl ACTH, Nl post-TSST ACTH	Severe BO: lower ACTH, cortisol response to TSST vs controls, whereas low BO: higher ACTH, cortisol responses vs controls
Schmaling [26]	2015	62	Healthy	ADAS	AUC, SCR	↓AUC, ↓SCR	31 couples studied, one of which had chronic fatigue (but not CFS)
Sveinsdottir [68]	2015	1150	Chronic Lombalgia	CFQ	DAC, CAR, SCR, NSC	Nl DAC, Nl CAR, Nl NSC, Nl SCR	**Colocar como SAUDÁVEL – porque é só lombalgia (increased CAR in Low back pain)
Powell [67]	2015	76	Multiple Sclerosis	CFQ	DAC, CAR, SCR	↓DAC, ↑CAR, Nl SCR	Sleep disorders excluded; adjusted for depressive symptoms; NSC not published. Multiple sclerosis had increased awakening cortisol and decreased CAR
Cruz [70]	2015	43	Breast cancer	BFI and CFQ	MSC, DHEA-S	Nl MSC, Nl DHEA-S	Pts undergoing ChTx included anthracyclines
Marchand [31]	2014	1043	H/B	Maslach	DAC, CAR, SCR, NSC	↑DAC, ↓CAR, ↑SCR, ↓NSC	Only BO; Groups of severe distress or depression not included
Aggarwal [30]	2014	227	Healthy	CFQ	MSC, NSC, 0.25 mg DST, DHEA-S	Nl MSC, Nl NSC, Nl 0.25 mg DST, ↓DHEA-S	Evaluation of chronic, widespread pain, chronic orofacial pain, chronic fatigue (but not CFS), irritable bowel syndrome
Tell D [71]	2014	130	Breast cancer	MFI	DAC, CAR, SCR, NSC	↓CAR, ↓SCR, ↑DAC, ↑NSC	Post-surgery breast cancer, regardless of ChTx. Not adjusted to sleeping patterns
Wolfram [32]	2013	53	H/B	Maslach	MSC, 1 mcg CST, DST/CRH test (1.5 mg DST + 100 mcg CRH)	Nl MSC, ↓post-1 mcg ACTH, Nl ACTH and cortisol DST-CRH	High over-commitment present blunted serum and salivary cortisol and ACTH responses to DST-CRH test
Klaassen [33]	2013	27	Healthy	MP (Beck & Luine, 2010) and Stress Tasks (Wang, 2006)	MSC, post-stress	Nl MSC, Nl post-stress	A complex test sequence was performed but not reproduced
Eek [34]	2012	581	Healthy	SOFI-20	DAC, CAR, NSC, SCR, MSC	Nl DAC, Nl CAR, Nl NSC, Nl SCR, Nl MSC	Women: reduced awakening, increased CAR, increased SCR; Men: increased awakening and reduced CAR – when fatigued
Sjors [35]	2012	247	H/B	DCSRD and SMBQ	DAC, 15 min CAR	Nl DAC, Nl 15 min CAR	
Rahman [54]	2011	30	CFS	Previous Dx – No questionnaire	MSC, SCR, NSC	Nl MSC, Nl SCR, Nl NSC	
Moya-Albiol [37]	2010	64	H/B	Maslach	DAC, CAR	Nl DAC, Nl CAR	
Kumari [38]	2009	4,299	Healthy	SF-36	DAC, CAR, NSC, SCR	↓DAC, ↓CAR, ↑NSC, ↓SCR	Adjusted for WC, BMI, sleep duration, CVD medication, depressive symptoms, smoking, alcohol intake provides Nl awakening but lower SCR
Osterberg [39]	2009	304	H/B	Maslach	DAC, CAR, NSC, SCR	Nl DAC, Nl CAR, ↓NSC, ↑SCR	0.5 mg DST was not compared to controls
Wingenfeld [41]	2009	279	H/B	Maslach and Maastricht	AUC, SCR	Nl AUC, Nl SCR	DAC and CAR not done; conclusions different from results. For AUC, Low BO: Nl, moderate: increased, severe: decreased
Rydstedt [40]	2009	76	Healthy	NRWS	DAC, NSC	Nl DAC, Nl NSC	
Papadopoulos [55]	2009	38	CFS	CFQ and SF-36	MSC, AUC, morning AUC, 0,5 mg DST	↑MSC, ↑AUC, ↑MAUC, Nl 0.5 mg DST	Data on absolute cortisol levels at each point not published. DST reduction evaluated by percent reduction.
Bay [72]	2009	75	Post traumatic brain injury	POMS	AUC	Nl AUC	Correlation between brain injury-related fatigue level and cortisol AUC. Basal and NSC results not reported; SCR not evaluated.
Sudhaus [73]	2009	43	Chronic Lombalgia	MFI	DAC, CAR, MAUC	↓CAR, Nl DAC, Nl MAUC (correlation between fatigue levels among low back pain subjects)	
Lindeberg [36]	2008	78	Healthy	SF-36	DAC, CAR, NSC, SCR	Nl DAC, ↓CAR, Nl NSC, ↓SCR	
Sertoz [42]	2008	72	H/B	Maslach	Basal and post 1.0 mcg DST cortisol	Nl basal cortisol and 1.0 mg DST	
Bellingrath [43]	2008	101	H/B	Maslach and Maastricht	DAC, CAR, NSC, SCR, 0.25 mg DST	Nl DAC, Nl CAR, Nl NSC, Nl SCR, ↓0.25 mg DST	
Nater [57]	2008	185	CFS	SF-36 and MFI	DAC, CAR, MAUC	Nl DAC, Nl CAR, ↓MAUC	
Torres-Harding [56]	2008	108	CFS	FSE	AUC, SCR	Nl AUC, Nl SCR	Multiple psychological tests performed. Data on NSC, basal and CAR not published.
Sonnenschein [45]	2007	42	H/B	Maslach	CAR, 0.5 mg DST, DHEA-S	Nl CAR, Nl 0.5 mg DST, Nl DHEA-S	Adjusted for depression, sleep quality. Awakening levels and each level graphics not available
Harris [44]	2007	44	Healthy	SF-36	DAC, CAR, NSC, SCR	Nl DAC, Nl CAR, Nl NSC, Nl SCR	Other aspects also correlated: complains, job stress and demand, QOL and coping. Adjusted for coffee and tobacco.
Langelaan [46]	2006	55	H/B	Maslach	DAC, CAR, 0.5 mg DST, DHEA-S	Nl DAC, Nl CAR, Nl 0.5 mg DST, Nl DHEA-S	Engaged work also compared and had stronger suppression in DST
Mommersteeg [47]	2006	109	Healthy	NC-WHO	DAC, CAR, 0.5 mg DST, SCR, AUC, NSC	Nl DAC, Nl CAR, Nl NSC, Nl SCR, Nl 0.5 mg DST, Nl AUC	
Barroso [74]	2006	40	HIV	HRFS	MSC, NSC	↓MSC, ↑NSC	
Jerjes [58]	2006	80	CFS	CFQ	UFC, TCM	↓UFC, Nl TCM	
Grossi [48]	2005	64	H/B	SMBQ	DAC, CAR	↓DAC, ↑CAR	Groups were high x moderate x low BO score; correlation was significant
Segal [59]	2005	40	CFS	No questionnaire	MSC, 1 mcg CST	↓ MSC, ↓1 mcg CST	DHEA-S collected only in CFS. No questionnaires used.
Jerjes [60]	2005	35	CFS	CFQ	MSC, SCR, NSC, AUC	↓MSC, ↓SCR, ↓AUC ↓, Nl NSC	
Bower [75]	2005	29	Breast cancer	SF-36	DAC, AUC, SCR, NSC	↑AUC, ↓SCR, Nl DAC, ↑NSC	Post-ChTx (regardless of time) complete cancer remission and exclusion of other disorders
McLean [76]	2005	55	Fybro-mialgia	SF-36	DAC, 60 min CAR, SCR, AUC, NSC	Nl DAC, Nl 60 min CAR, Nl SCR, Nl AUC, Nl NSC (correlation between fatigue levels among FMG subjects)	FMG subjects presented Nl DAC and CAR, as controls.
Roberts [62]	2004	92	CFS	CFQ and SF-36	DAC, CAR, MAUC	Nl DAC, ↓CAR, ↓MAUC	
Crofford [61]	2004	72	CFS/FMG	POMS	ACTH, MSC, SCR, NSC, AUC	Nl ACTH, Nl SCR, Nl NSC, ↓AUC, Nl MSC	Tests performed in: CFS, FMG and CFS + FMG; FMG w/o fatigue had Nl AUC and increased BMC levels
Moch [50]	2003	16	H/B	Maslach	UFC, DHEA-S, ACTH, MSC	↓UFC, Nl DHEA-S, Nl ACTH, ↓MSC	Only women; longitudinal evaluation – Nl initial cortisol.
De Vente [49]	2003	45	H/B	Maslach	DAC, MSC, post-TSST	↑DAC, ↑MSC, Nl post-TSST	
Gaab [63]	2002	42	CFS	MFI	DAC, CAR, SCR, 0.5 mg DST	↓0.5 mg DST, Nl AUC, Nl CAR, Nl DAC, Nl SCR, Nl NSC	CAR also performed at 15, 45 and 60 min.
Dekkers [77]	2000	53	Rheumatoid Arthritis	MFI	DAC, CAR, SCR, AUC	Nl AUC, Nl SCR Nl, ↓DAC, ↑CAR	5/25 subjects with RA taking prednisone (5–10 mg/d); RS subjects had smaller SCR, increased AM cortisol and decreased CAR. 15 and 45 min CAR also performed.
Melamed [51]	1999	111	H/B	SMBQ and Maastricht	MSC and 4 PM cortisol	↑MSC, Nl 4 PM cortisol	
Pruessner [52]	1999	66	Healthy	Maslach	DAC, CAR, 0.5 mg DST	↓DAC, ↓CAR, ↓0.5 mg DST	15 min and 60 min CAR also performed
Strickland [65]	1998	74	CFS	Not specified/detailed	MSC, NSC	↓NSC, Nl MSC	Adjusted for depression
Young [66]	1998	45	CFS	NC-WHO	UFC, SCR, MSC, AUC	Nl UFC, Nl SCR, Nl MSC, Nl AUC	
Scott [64]	1998	28	CFS	Not specified (not detailed)	MSC, ACTH, 100 mcg CRH cortisol stimulation	Nl MSC, Nl ACTH, CRH stim test: ↓cortisol, ↓ACTH	
Raikkonen [53]	1996	22	Healthy	Not assessed	1 mcg CST, DST (non specified), OGTT, MSC, ACTH, cortisol/ACTH ratio	↑Cortisol/ACTH ratio; ↑CST, Nl DST, Nl OGTT, Nl MSC, Nl ACTH	Full article not assessed – not in PUBMED or other database

Questionnaires: *SMBQ* shirom-melamed burnout questionnaire, *BFI* brief fatigue inventory, *CFQ* chalder fatigue questionnaire, *Maslach* maslach burnout inventory, *SF*-*36* short form health survey 36, *NC* *-* *WHO* neurasthenia criteria, *DCSRD* diagnosis criteria of stress-related exhaustion disorder, *SEQ* stress-energy questionnaire, *ADAS*, abbreviated dyadic adjustment scale, *MFI* multidimensional fatigue inventory, *FAQ* fatigue assessment questionnaire, *MP* memory performance, *POMS* profile of mood states, *Stress Tasks*, *FSE* fatigue severity scale, *SOFI* Swedish occupational fatigue inventory, *Maastricht* Maastricht vital exhaustion questionnaire, *NRWS* need for recovery from work scale, *HRFS* HIV-related fatigue scale, *WC* waist circumference

Other abbreviations: *CFS* chronic fatigue syndrome, *H* */* *B* healthy/burnout, *24 h*-*UFC* 24-h urinary free cortisol, *FMG* fibromyalgia; ↑: Increased or elevated; ↓: Decreased or reduced; →: Unchanged; *Nl*: Normal

### Study characteristics

Among the *58* studies included, *33 (56.9 % of the selected studies)* were performed in healthy subjects [21–53], since we considered “burnout” not an actual disorder but instead a stressful condition presented by some groups of health workers. Despite the several studies describing cortisol impairment in Chronic Fatigue Syndrome (CFS), only 13 *(22.4 %)* studies performed an actual assessment of the hypothalamic–pituitary–adrenal (HPA) axis [54–66]. Twelve studies (20.7 %) were found in which tests for cortisol profiling were performed for other diseases [67–77]. However, for analysis purposes, one study [69] was divided into two studies as it performed two distinct protocols at different moments. Among these, five were done performed in patients with a diagnosis of breast cancer who had undergone or were undergoing chemotherapy. One study tested patients with fibromyalgia, two studies compared patients with chronic lower pain, one with rheumatoid arthritis, one with post brain injury, two with multiple sclerosis, and one involved patients with human immunodeficiency virus (HIV) and CFS. One study evaluated both patients with fibromyalgia and patients with CFS in different groups.

The median number of tested subjects in the 58 studies was 72 (range: 16–4,299). The median numbers of participants in articles involving healthy individuals, patients with CFS, or patients with other diseases were 76 (16–4,299), 45 (28–185), and 65 (29–1150), respectively. The largest number of healthy subjects included groups of workers whose cortisol results were compared to exhaustion and fatigue status, in an attempt to discriminate correlations between cortisol and energy levels. One study involving 4,299 individuals was responsible for more subjects than the sum of all the other studies.

### Methods used to evaluate fatigue in the general study population

Some authors utilized more than one method to compare the different patients and were included in multiple groups. A summary of all the methods used to assess fatigue, and their results, is shown in Table 2. Among the 58 studies, 27 (46.6 %) utilized the Cortisol Awakening Response (CAR) to assess the HPA axis. This method is based on previous studies [77–81] that indicate cortisol levels rise by 50 % on average within 30 min of waking as a physiological response to stay alert, with a blunted CAR resulting in fatigue symptoms. For the CAR, salivary cortisol is collected immediately on waking (t = 0) and again 30 min later (t = 30), and the difference (delta cortisol) between the two measurements are analyzed. Among the 27 studies that employed CAR, fourteen (51.9 %) showed a normal response, nine (23.3 %) had a diminished delta cortisol, and four (14.8 %) demonstrated an increased delta cortisol.

**Table 2:** Assessed methods and results of all selected studies (*N =* 58)

Procedure (*)	Number of studies (% of total)	Not different (%)	Decreased (%)	Increased (%)
DAC	29 (50.0 %)	19 (65.5 %)	6 (20.7 %)	4 (13.8 %)
CAR	27 (46.6 %)	14 (51.9 %)	9 (33.3 %)	4 (14.8 %)
SCR	26 (44.8 %)	16 (61.5 %)	7 (26.9 %)	3 (11.5 %)
MSC	22 (37.9 %)	14 (63.6 %)	4 (18.2 %)	4 (18.2 %)
NSC	22 (37.9 %)	13 (59.1 %)	3 (13.6 %)	6 (27.3 %)
AUC	13 (22.4 %)	8 (61.5 %)	3 (23.1 %)	2 (15.4 %)
DST	9 (15.5 %)	6 (66.7 %)	3 (33.3 %)	-
DHEA-S	6 (10.3 %)	4 (66.7 %)	2 (33.3 %)	-
ACTH	6 (10.3 %)	5 (83.3 %)	-	1 (16.7 %)
MST	5 (8.6 %)	4 (80.0 %)	1 (20.0 %)	-
UFC	3 (5.2 %)	1 (33.3 %)	2 (66.7 %)	-
CST	3 (5.2 %)	-	2 (66.7 %)	1 (33.3 %)
MAUC	3 (5.2 %)	-	2 (66.7 %)	1 (33.3 %)
CAR 60 min	2 (3.4 %)	2 (100 %)	-	-
ACTH MST	2 (3.4 %)	2 (100 %)	-	-
4 PM cortisol	1	1	-	-
DST + CRH cortisol	1	1	-	-
DST + CRH ACTH	1	1	-	-
CAR 15 min	1	1	-	-
TCM	1	1	-	-
DHEA-S MST	1	-	1	-
CRST cortisol	1	-	1	-
CRST ACTH	1	-	1	-
OGTT cortisol	1	-	-	1
Cortisol/ACTH ratio	1	-	-	1

Legends: (*): *DAC* direct awakening cortisol, *CAR* cortisol awakening response, *SCR* salivary cortisol rhythm, *MSC* morning serum (& salivary) cortisol, *NSC* night salivary cortisol, *AUC* area under-the-curve (Estimated Cortisol Release), *DST* dexamethasone suppression test, *DHEA* *-* *S* dehydroepiandrosterone sulfate, *ACTH* adrenocorticotropic hormone, *MST* mental stress test, *UFC* 24 h-urinary free cortisol, *CST* cosyntropin stimulation test, *MAUC* morning area under-the-curve (morning estimated cortisol release), *CRH* corticotropin releasing hormone, *TCM* total urinary cortisol metabolites, *CRST* corticotropin releasing stimulation test (?), *OGTT* oral glucose tolerance test

Another method that became widely used to evaluate exhaustion/burnout/fatigue states is the salivary cortisol rhythm (SCR), which evaluates the change in cortisol levels between morning, afternoon, and late night. A total of 26 studies evaluated SCR (44.8 %). Some heterogeneity in the method was found between studies, but in general, salivary cortisol was collected at 8 AM, 4 PM, and 10–11 PM. While the SCR is considered as another fatigue marker [82, 83], like the CAR, there is no justification for considering this as an etiology for “adrenal fatigue”. Sixteen (61.5 %) studies showed no difference between fatigued and control patients, whereas seven (26.9 %) demonstrated an impaired decrease in the circadian SCR. The remaining three (11.6 %) studies disclosed a more pronounced decrease in cortisol level.

The direct awakening cortisol (DAC) level, collected at the exact moment of waking, was used in 29 studies (50.0 %). Unlike CAR, DAC reflects sleep quality rather than being a possible identifying factor of fatigue [84–86], even though a poor quality sleep plays an important role in the fatigue process [87–89]. In studies that employed DAC, inconsistent results were observed: normal results were found in nineteen (65.5 %) studies, elevated levels were shown in four (13.8 %), and reduced levels in six (20.7 %).

The DAC, CAR and SCR methods were by far the most commonly elected ones for examining the correlation between cortisol profile and fatigue status. However, a few other studies analyzed other aspects of cortisol release.

The dexamethasone (Dex) suppression test (DST) was also used in nine (15.3 %) studies. The DST identifies autonomous hypercortisolism, as cortisol production is normally suppressed by Dex. DSTs have also been used to investigate hypocortisolism, based on the supposed assumption that it promotes “oversuppression” of cortisol in low cortisol states, indicating that lower levels of cortisol would disclose a more prolonged suppression than controls [55, 90–93], although many studies do not show correlation between DST and fatigue [47, 55, 94, 95]. In six studies, a lower Dex dose (0.5 mg) was used in an attempt to improve the test sensitivity. Among these, four studies (66.7 %) showed the same results for both groups, whereas in two others (33.3 %), the test resulted in lower and prolonged suppression of cortisol levels in fatigued subjects. Moreover, an even lower Dex dose (0.25 mg) was performed in two studies and resulted in reduced cortisol in one study and normal levels in the group with exhaustion. In one study, Dex dose was not specified, but levels were not different among exhausted and control groups. As a whole, the DST was used in nine studies, and no significant differences were observed between fatigued and non-fatigued groups in six of these studies (66.7 %), whereas reduced levels were observed in three studies (33.3 %).

Adrenocorticotropic hormone (ACTH) is a pituitary peptide hormone that stimulates cortisol production by the adrenocortical zona fasciculata. Elevated ACTH occurs early in primary adrenal insufficiency, whereas inappropriate (normal) ACTH levels in the presence of low serum cortisol are found in secondary adrenal failure. Although, normal ACTH levels with normal cortisol levels does not exclude the possibility of relative adrenocortical failure. Six (10.3 %) studies employed the morning ACTH levels to compare fatigued and non-fatigued patients; no significant differences for ACTH, as well as for cortisol, were found in five studies (83.3 %), meanwhile one showed elevated ACTH levels in burnout patients (16.7 %).

On the other hand, three studies (5.2 %) used the low-dose cosyntropin (a synthetic _1-24_ACTH) stimulation test (CST), in which 1 μg of cosyntropin is used instead of the classic 250 μg dose, based on the premise that the CST is more accurate and sensitive for verifying the adrenocortical cortisol reserve [96], even though most findings indicate that both doses have similar accuracy [97, 98]. Surprisingly, one of three (33.3 %) studies disclosed a paradoxically higher cortisol increase compared to controls, while in two (66.7 %) lower levels were observed. Conversely, impaired cortisol and ACTH responses was observed in the fatigued group in a single study in which corticotropin-releasing hormone (CRH) was used to stimulate the HPA axis.

Three (5.2 %) studies measured 24 h-urinary free cortisol (UFC) in an attempt to correlate cortisol excretion rates with intensity of fatigue. Although the 24 h-UFC reflects the total cortisol produced per day, it was initially conceived to investigate cortisol excess syndromes, although diminished levels could hypothetically imply subnormal adrenal function, despite of lack of any evidence. One of these studies (33.3 %) found no correlation between 24 h-UFC and energy status, whereas two studies (66.7 %) showed reduced values in fatigued patients.

Thirteen studies (22.4 %) estimated total cortisol release (AUC) by calculating the areas under the curves for the whole day salivary cortisol collection by using three or more daily salivary cortisol levels over four or more days. Assessment of the total 24 h cortisol release by this method would complement the SCR, since the lack of the expected decrease throughout the day observed in some studies can be due either to a non-elevated morning serum cortisol (MSC) level or to a full day elevated cortisol, although three daily levels of cortisol is probably too few for a minimally precise AUC; herein, findings are conflicting. AUC was elevated in two (15.4 %) studies, normal in eight (61.5 %), and reduced in another three (23.1 %).

Twenty-two studies (37.9 %) compared baseline MSC between controls and fatigued patients; traditionally [98], this is the initial cortisol assessment to investigate possible hypocortisolism. Basal MSC was not different between individuals in fourteen (63.6 %) of these studies, was significantly reduced in fatigued patients in three (23.1 %), and was elevated in two (15.4 %).

Twenty-two articles (37.9 %) correlated late night salivary cortisol (11 PM NSC) and fatigue status. The NSC was initially validated to assess cortisol excess, as physiologically, one expects lower cortisol levels at the end of the day; although, NSC has been extended to investigate hypocortisolism in these studies, despite of lack of validation. Three studies (13.6 %) showed a lower cortisol level in fatigued subjects compared to controls, thirteen (59.1 %) found no differences, and six (27.3 %) showed increased levels in fatigued subjects.

Six studies (10.3 %) investigated the correlation between dehydroepiandrosterone sulfate (DHEA-S) levels and fatigue status. Reduced DHEA-S levels are usually found in hypocortisolism and are a potential marker of fatigue, although there is still not enough evidence to corroborate this affirmation. Four studies (66.7 %) found no correlation with DHEA-S, whereas two (33.3 %) found lower levels in chronic exhausted patients.

The morning estimated total cortisol release (MAUC) is obtained by calculating the area under the curves for the period between the awakening moment and 1 hour later, and is based on determining three or more salivary cortisol levels during this period of the day, although this method has also not been validated by any indexed study. A total of four studies (6.9 %) among the selected studies reported the MAUC. Two of these studies (50.0 %) showed reduced MAUC levels in fatigued subjects, one demonstrated increased results (25.0 %), and one demonstrated no differences (25.0 %).

Mental stress tests (MST) have been performed in some studies in order to identify possible differences in cortisol and ACTH release between fatigued and non-fatigued individuals. The most employed test was the Trier Social Stress Test (TSST), which has been already validated as a stress trigger test [99–102], and requires complete HPA axis integrity for a proper response. Other types of MSTs have also been proposed and validated [103, 104]. MSTs were performed in five different studies in order to correlate cortisol and ACTH responses and burnout status. No difference was seen in four studies (80.0 %), whereas in one (20.0 %), cortisol and ACTH responses were impaired in exhausted individuals.

Some other tests were performed in a smaller number of the selected studies, as follows: two studies performed a 60 min CAR (both showed normal results among fatigued and non-fatigued subjects); one study performed a 15 min CAR (and showed normal results); two studies performed the ACTH MST (both used the TSST and found normal results); one study performed the DHEA-S MST (which also used the TSST and demonstrated normal results); one study performed the cortisol post Oral Glucose Tolerance Test (OGTT) (and found no differences among fatigued and non-fatigued subjects); one study calculated cortisol/ACTH ratio (and found an increased ratio among exhausted subjects); one study evaluated the 4 PM cortisol level (and found no significant differences between exhausted subjects and controls); one study used 1.5 mg-Dex followed by 0.1 mg-CRH to stimulate cortisol and ACTH (and showed normal responses); one study stimulated ACTH and cortisol with 0.1 mg of CRH (and found reduced levels of both hormones in fatigued subjects compared to controls); and finally, one study evaluated the multiple urinary cortisol metabolites and calculated the Total Cortisol Metabolites (TCM) (and found no differences between fatigued subjects and controls).

Finally, we were not able to find studies in which the gold standard test for assessing the integrity and functionality of the HPA axis—the insulin tolerance test (ITT)—were performed. The same was true for the lipopolysaccharides (LPS) stimulation test. Both tests stimulate hypothalamic CRH secretion, leading to a complete evaluation of the HPA axis.

### Fatigue in burnout syndrome

Burnout syndrome or clinical burnout, or simply “burnout”, refers to a decrease in the cognitive functions, emotional exhaustion, and physical fatigue that is triggered by stressful situations associated with excessive working [105]. However, there is no pathognomonic marker for burnout [105]. For practical purposes, we considered non-CFS burnout patients as “healthy”, as burnout is yet to be considered a disease and its characterization is still heterogeneous. A summary of the performed methods and their respective results in non-CFS burnout/healthy patients [21–53] are shown in Table 3. Assessment of the HPA axis integrity in burnout patients (at the pituitary and hypothalamic levels) has not been determined.

**Table 3:** Studies in Burnout syndrome and healthy subjects (*N =* 33): Methods of assessment and respective results

Procedure	Number of studies (% of total)	Not different (%)	Decreased (%)	Increased (%)
DAC	17 (51.5 %)	10 (58.8 %)	4 (23.5 %)	3 (17.7 %)
CAR	16 (48.5 %)	9 (56.2 %)	6 (37.5 %)	1 (6.3 %)
MSC	12 (36.4 %)	8 (66.7 %)	1 (8.3 %)	3 (25.0 %)
SCR	12 (36.4 %)	7 (58.3 %)	3 (25.0 %)	2 (16.7 %)
NSC	10 (30.3 %)	7 (70.0 %)	2 (20.0 %)	1 (10.0 %)
DST	7 (21.2 %)	5 (71.4 %)	2 (28.6 %)	-
MST	5 (15.2 %)	4 (80.0 %)	1 (20.0 %)	-
DHEA-S	5 (15.2 %)	3 (60.0 %)	2 (40.0 %)	-
ACTH	4 (10.1 %)	3 (75.0 %)	-	1 (25.0 %)
AUC	3 (9.1 %)	2 (66.7 %)	1 (33.3 %)	-
ACTH MST	2 (6.1 %)	2 (100 %)	-	-
CST	2 (6.1 %)	-	1 (50 %)	1 (50 %)
4 PM cortisol	1	1	-	-
DST + CRH cortisol	1	1	-	-
DST + CRH ACTH	1	1	-	-
CAR 15 min	1	1	-	-
CAR 60 min	1	1	-	-
DHEA-S MST	1	-	1	-
UFC	1	-	1	-
OGTT cortisol	1	-	-	1
Cortisol/ACTH ratio	1	-	-	1

Legends: (*): *DAC* direct awakening cortisol, *CAR* cortisol awakening response, *MSC* morning serum (& salivary) cortisol, *SCR* salivary cortisol rhythm, *NSC* night salivary cortisol, *DST* dexamethasone suppression test, *MST* mental stress test, *DHEA* *-* *S* dehydroepiandrosterone sulfate, *ACTH* adrenocorticotropic hormone, *AUC* area under-the-curve (estimated cortisol release), *CST* cosyntropin stimulation test, *CRH* corticotropin releasing hormone, *UFC* 24 h-urinary free cortisol, *OGTT* oral glucose tolerance test

### Fatigue in chronic fatigue syndrome

CFS is a diagnosis used for patients who present severe fatigue for more than six months, not explained by any hormonal, metabolic, inflammatory, or other disorders. Correlations between CFS and the HPA axis have been studied [54–66] and the results are shown in Table 4.

**Table 4:** Studies in Chronic Fatigue Syndrome (*N =* 13): Methods of assessment and respective results

Procedure	Number of studies (% of total)	Not different (%)	Decreased (%)	Increased (%)
MSC	8 (61.5 %)	5 (62.5 %)	2 (25.0 %)	1 (12.5 %)
SCR	6 (46.2 %)	5 (83.3 %)	1 (16.7 %)	-
AUC	6 (46.2 %)	3 (50.0 %)	2 (33.3 %)	1 (16.7 %)
NSC	5 (38.5 %)	4 (80.0 %)	1 (20.0 %)	
DAC	3 (23.1 %)	3 (100.0 %)	-	-
CAR	3 (23.1 %)	2 (66.7 %)	1 (33.3 %)	-
ACTH	2 (15.4 %)	2 (100.0 %)	-	-
DST	2 (15.4 %)	1 (50.0 %)	1 (50.0 %)	-
UFC	2 (15.4 %)	1 (50.0 %)	1 (50.0 %)	-
CST	1 (7.7 %)	-	1 (100.0 %)	-

Legends: (*): *MSC* morning serum (& salivary) cortisol, *SCR* salivary cortisol rhythm, *AUC* area under-the-curve (estimated cortisol release), *NSC* night salivary cortisol, *DAC* direct awakening cortisol, *CAR* cortisol awakening response, *ACTH* adrenocorticotropic hormone, *DST* dexamethasone suppression test, *UFC* 24 h-urinary free cortisol, *CST* cosyntropin stimulation test

### Fatigue in other disorders

Complaints regarding fatigue not entirely explained by the underlying pathophysiology of the disease have been observed in patients suffering from other disorders, such as chronic low back pain [106, 107], breast cancer survivors [108–110], and HIV [111, 112]. Therefore, the role of the HPA axis in the etiology of fatigue in these subjects has been analyzed [67–77] and the findings are presented in Table 5.

**Table 5:** Studies in Other Disorders (*N =* 12): Methods of and respective results

Procedure	Number of studies (% of total studies)	Not different (%)	Decreased (%)	Increased (%)
DAC	9 (75.0 %)	6 (66.7 %)	2 (22.2 %)	1 (11.1 %)
SCR	8 (66.7 %)	4 (50.0 %)	3 (37.5 %)	1 (12.5 %)
CAR	8 (66.7 %)	3 (37.5 %)	2 (25.0 %)	3 (37.5 %)
NSC	7 (58.3 %)	2 (28.6 %)	-	5 (71.4 %)
AUC	4 (33.3 %)	3 (75.0 %)	-	1 (25.0 %)
MSC	2 (16.7 %)	1 (50.0 %)	1 (50.0 %)	-
DHEA-S	1 (8.3 %)	1 (100.0 %)	-	-

Legends: (*): *DAC* direct awakening cortisol, *SCR* salivary cortisol rhythm, *CAR* cortisol awakening response, *NSC* night salivary cortisol, *AUC* area under-the-curve (estimated cortisol release), *MSC* morning serum (& salivary) cortisol, *DHEA* *-* *S* Dehydroepiandrosterone sulfate

### Questionnaires for fatigue assessment

Among all studies included in this review, nineteen different types of questionnaires and scores were reported. The most commonly used were: the Maslach Burnout Inventory (MBI, *n =* 15), SF-36 (*n =* 9), the Chalder Fatigue Scale (CFS, *n =* 8), the General Fatigue Scale of the Multidimensional Fatigue Inventory (MFI, *n =* 6) and the Shirom Melamed Burnout Questionnaire (*n =* 6). In ten studies, more than one type of survey was performed. In four studies, the methods to assess fatigue were not specified or assessed. A summary of the assessed questionnaires is shown in Table 6.

**Table 6:** Assessed questionnaires employed in the selected studies (*N =* 58)

Questionnaire	General	Healthy/Burnout	CFS	Other diseases
*Maslach* Burnout Inventory	15 (25.7 %)	15	-	-
*SF*-*36* *-*Short Form Health Survey 36	9 (15.5 %)	4	3	2
*CFQ* *-*Chalder Fatigue Questionnaire	8 (13.8 %)	1	4	3
*SMBQ* - Shirom-Melamed Burnout Questionnaire	6 (10.3 %)	6	-	-
*MFI* *-*Multidimensional Fatigue Inventory	6 (10.3 %)	-	2	4
*DCSRD*: Diagnosis criteria of stress-related exhaustion disorder	3 (5.2 %)	3	-	-
*Maastricht* Vital Exhaustion Questionnaire	2 (3.4 %)	2	-	-
*FAQ* *-*Fatigue Assessment Questionnaire	2 (3.4 %)	-	-	2
*NC*-*WHO* *-*Neurasthenia Criteria	2 (3.4 %)	1	1	-
*POMS* *-*Profile of Mood States	2 (3.4 %)	-	1	1
*SOFI* *-*Swedish Occupational Fatigue Inventory	1	1	-	-
*ADAS* *-*Abbreviated Dyadic Adjustment Scale	1	1	-	-
*SEQ* *-*Exclusion with Stress-Energy Questionnaire	1	1	-	-
*MP* *-*Memory performance	1	1	-	-
*Stress Tasks*	1	1	-	-
*NRWS* - Need for Recovery from Work Scale	1	1	-	-
*FSE* *-*Fatigue Severity Scale	1	-	1	-
*HRFS* *-*HIV-related Fatigue Scale	1	-	-	1
*BFI*: Brief Fatigue Inventory	1	-	-	1
More than one questionnaire	10 (17.2 %)	6	3	1
Stress tests	5 (8.6 %)	5	-	-
Not specified	4 (6.9 %)	-	4	

Legends: *Maslach* maslach burnout inventory, *SF-36* short form health survey 36, *CFQ* chalder fatigue questionnaire, *SMBQ* shirom-melamed burnout questionnaire, *MFI* multidimensional fatigue inventory, *DCSRD* diagnosis criteria of stress-related exhaustion disorder, *Maastricht* Maastricht vital exhaustion questionnaire, *FAQ* fatigue assessment questionnaire, *NC* *-* *WHO* neurasthenia criteria, *POMS* profile of mood states, *SOFI* Swedish occupational fatigue inventory, *ADAS* abbreviated dyadic adjustment scale, *SEQ* stress-energy questionnaire, *MP* memory performance, *Stress Tasks*; *NRWS* need for recovery from work scale, *FSE* fatigue severity scale, *HRFS* HIV-related fatigue scale, *BFI* brief fatigue inventory

## Discussion

Theories on adrenal impairment as the genesis for fatigue are tempting, as they allow for a treatable condition. Despite the widespread use of the term “adrenal fatigue” by the general media and certain health practitioner groups, in this systematic review, only ten citations [113–122] were found with this exact expression, and they were all only descriptive and did not perform any test regarding the HPA axis and “adrenal fatigue”. Studies that tried to correlate the HPA axis and fatigue states used the term “burnout” instead of “adrenal fatigue” to denote adrenal depletion. Therefore, a distinction between the “general information” and the actual scientific literature regarding this condition is evident. First, this suggests that the terminology of a hypothetical adrenal depletion should be normalized, with a suitable name given for the purported condition, as “adrenal fatigue” has been already been stigmatized and lacks proper scientific support. Second, methodology employed to evaluate the proposed correlation between fatigue and adrenal function should be standardized among physicians and medical associations that claim for the existence of adrenal impairment in patients with fatigue before evident clinical hypocortisolism manifests, in order to strength eventual evidence, in case one finds actual and proper causal correlation.

No confirmed methods of clinical screening for AF are available. Indeed, the popular questionnaire developed by Dr. Wilson and published in the first book exclusively dedicated to the description of this supposedly disease [6] has not been cited in any indexed databases. Another theory, the “Thompson cortisol hypothesis” [123], suggests that cortisol is responsible for yawning and fatigue; however, again, no studies that tested this theory have been published in indexed journals. Validated surveys have been used in studies that investigate fatigue states, but they were not correlated with proper cortisol assessment methods. The TSST is the only survey to have enough credibility to be officially tested and standardized as a trigger of stress [99–102].

Functional tests are the only methods to assess adrenal cortisol production endorsed by endocrinology societies [97]. Although, the ITT is considered the gold standard test to evaluate the entire HPA axis, neither the ITT (or the similar LPS stimulation test) was performed in any studies investigating the correlation between fatigue states and adrenocortical function. Moreover, we generally found conflicting data using most of the functional tests when trying to differentiate exhausted, fatigued, and burnout individuals from healthy patients. For example, using the low-dose CST, we found an unexpected increase in cortisol levels in fatigued subjects in the selected studies. This may have been perhaps the result of a relative secondary adrenal insufficiency, which leads to an amplified adrenal cortisol response due to an upregulation of ACTH receptors, but this sounds unjustifiable since the lack of continuous stimulation of the adrenal cortices would cause atrophy, rendering them non-responsive to a low- (and even high) dose of cosyntropin stimulation in the long run. Regardless of the theoretical explanation, CST has shown to be not a good marker of fatigue. Similarly, ACTH levels were also poorly studied and did not show significant correlations in most fatigued subjects. In addition, despite its lack of standardization, the DST was performed in nine studies, but conflicting results invalidated attempts to establish this as a new marker for fatigue states. Moreover, the 24 h-UFC has been shown to be so far inaccurate for investigation of adrenal impairment. Findings were also contradictory in the six studies that calculated cortisol AUC as well as in the four studies that performed MAUC. Therefore, the above methods cannot be used to differentiate fatigued from non-fatigued individuals.

In this review, we also examined whether cortisol markers can be used to assess cortisol impairment. The results of our review indicate that the three major tests (CAR, DAC and SCR) used to identify the underlying causes of the fatigue/exhaustion state failed to do so, since they were unable to demonstrate significant differences or proper causality. CAR and DAC frequently showed inconsistent results in studies that used heterogeneous groups of subjects. CAR and DAC are not necessarily indicatives of the etiology and pathogenesis of the fatigue status, since both can be consequences of other disorders, such as sleep disturbances. Indeed, a recent study [124] was the first to use CAR as a marker of improvement of burnout syndrome, which reinforces the use of this method for monitoring the consequences of fatigue states, but not for its etiology [77–81].

With regards to the SCR, the results may be misleading if they are not analyzed together with the total 24 h cortisol release. This is because a non-physiological blunted rhythm can be due either to an impairment of the lowering cortisol trend throughout the day or due to a lower morning cortisol level. Despite this, studies that evaluated total 24 h cortisol by measuring serial salivary cortisol levels also showed conflicting findings. Our systematic review corroborates another systematic review [83] that shows inconsistency regarding measuring methods among across different randomized controlled trials. Similarly, baseline MSC and NSC were poor markers of fatigue status as it failed to reveal any differences in burnout/exhaustion/fatigue patients compared to healthy subjects.

Adrenal size could be considered another marker of adrenal activity, as hypertrophic/hyperplastic adrenal glands could be the result of an ACTH over-stimulation by the pituitary, as seen in subjects exposed to chronic stress [125, 126], whereas a diminished or atrophic gland may reflect adrenal insufficiency at any level of the HPA axis [98]. However, not a single study could be identified in which the adrenal size has been checked in fatigued or exhausted patients. Similarly, although DHEA-S could also be a potential marker for adrenal atrophy or dysfunction, is still uncertain whether it plays any pathophysiological role in fatigue. Finally, none of the abovementioned methods were accurate markers of fatigue, nor could they be correlated with the HPA axis dysfunction as an etiology of fatigue.

It is also important to note that once adrenal impairment is confirmed using any of these tests, the etiology should also be elucidated. As the HPA axis can be affected by several chronic and/or metabolic disorders, other primary conditions must be excluded before intrinsic disorders of the HPA axis are deemed responsible. Typical differential diagnosis of “adrenal fatigue” and related states are: (1) sleep obstructive apnea syndrome; (2) adrenal insufficiency; (3) mental illnesses; (4) excessive working (overwork); (5) night-shift workers; (6) other hormonal deficiencies; (7) liver and kidney dysfunctions; (8) heart conditions; (9) chronic pulmonary obstructive disease; (10) autoimmune diseases.

Although conflicting data were reported, patients with CFS tend to have a normal cortisol profile, and the abnormalities found can be typically be explained by a poor quality sleeping patterns. Therefore, health providers should not be concerned about adrenal function in CFS subjects once they had been already excluded to other conditions prior to the diagnosis of CFS. Similarly, studies investigating patients with the burnout syndrome were greatly inconsistent So far, HPA axis tests should not be used as markers for burnout syndrome by health practitioners. Similar conclusions can be drawn for the use of HPA axis tests as markers for fibromyalgia and other chronic diseases, which tend to demonstrate inconsistent findings, whereas studies that were performed in breast cancer subjects tended to show depletion of cortisol levels; however, studies in breast cancer were performed while administering chemotherapy, which can introduce a confounding bias.

Therefore, based on our current knowledge, cortisol tests should not yet be used in clinical practice for examining any condition, except if adrenal impairment is suspected. Moreover, glucocorticoid therapy should be avoided in patients, as it can increase the risk of cardiovascular disease or osteoporosis, even in low doses.

### Limitations

Some limitations of this review include: (1) our inability to perform a meta-analysis due to heterogeneity of the study design; (2) the descriptive nature of most studies, and the reporting of a condition that has not been scientifically proven without adding new data nor providing solid arguments; (3) the fact that most studies were published in low impact journals; (4) the inadequate and poor quality assessment of fatigue; (5) the use of an unsubstantiated methodology in terms of cortisol assessment; (6) the lack of concern regarding validated adrenal assessment (as endorsed by endocrinologists); (7) false premises leading to an incorrect sequence of thinking and research direction; and, (8) inappropriate/invalid conclusions regarding causality and association between different information, in particular, whether any abnormalities would be a marker or a potential target for treatment.

### Final discussions

Our results corroborate an Endocrine Society warning statement regarding adrenal fatigue (1), as saying that “adrenal fatigue is not a real medical condition”. While a recent systematic review on burnout was published (109) that implicated some HPA dysfunctions as markers or triggers of burnout, there were important bias selection regarding the articles chosen. Therefore, we recommend that for further prospective studies aiming to correlate fatigue, exhaustion, or burnout status with impairment of the HPA axis, an ITT or a 250 μg CST should be performed to evaluate the adrenocortical ability to release cortisol, measurements of ACTH, DHEA-S, and corticosterone (an intermediate steroid product that is impaired earlier than cortisol [127]), the adoption of the most validated questionnaires, particularly Maslach Burnout Inventory, the Chalder Fatigue Scale, SF-36 or the General Fatigue Scale of the Multidimensional Fatigue Inventor, and considering different study populations, including: (a) healthy subjects; (b) burnout healthy subjects; (c) subjects with overtraining syndrome; (d) subjects post-chemotherapy; (e) subjects with CFS; and (f) subjects with fibromyalgia.

In addition, we do not recommend the use of the many methods reported in the articles evaluated in this systematic review, as they are not accurate to determine whether a patient has or has not adrenal failure.

The answer to whether “adrenal fatigue” or depletion exists or not may not be simple, but different answers can be offered according to the presence of an underlying disease. However, so far, there is no substantiation to show its existence.

## Conclusion

To our knowledge, this is the first systematic review made by endocrinologists to examine a possible correlation between the HPA axis and a purported “adrenal fatigue” and other conditions associated with fatigue, exhaustion or burnout. So far, there is no proof or demonstration of the existence of “AF”. While a significant number of the reported studies showed differences between the healthy and fatigued groups, important methodological issues and confounding factors were apparent. Two concluding remarks emerge from this systematic review: (1) the results of previous studies were contradictory using all the methods for assessing fatigue and the HPA axis, and (2) the most appropriate methods to assess the HPA axis were not used to evaluate fatigue. Therefore, “AF” requires further investigation by those who claim for its existence.

## Abbreviations

24 h UFC: 24-h urinary free cortisol
ACTH: Adrenocorticotropic hormone
ADAS: Abbreviated dyadic adjustment scale
AF: Adrenal fatigue
AUC: Estimated cortisol release (area under the curve)
BFI: Brief fatigue inventory
CAR: Cortisol awakening response
CFQ: Chalder fatigue questionnaire
CFS: Chronic fatigue syndrome
CST: Cosyntropin stimulation test
DAC: Direct awakening cortisol
DCSRD: Diagnosis criteria of stress-related exhaustion disorder
DHEA-S: Dehydroepiandrosterone sulfate
DST: Dexamethasone suppression test
FAQ: Fatigue assessment questionnaire
FMG: Fibromyalgia
FSE: Fatigue severity scale
H/B: Healthy/Burnout
HRFS: HIV-related fatigue scale
Maastricht: Maastricht vital exhaustion questionnaire
Maslach: Maslach burnout inventory
MFI: Multidimensional fatigue inventory
MP: Memory performance
MSC: morning serum cortisol (& salivary)
MST: Mental stress tests
NC-WHO: Neurasthenia criteria
NRWS: Need for recovery from work scale
NSC: Night salivary cortisol
POMS: Profile of mood states
SCR: Salivary cortisol rhythm
SEQ: Exclusion with stress-energy questionnaire
SF-36: Short form health servey 36
SMBQ: Shirom-melamed burnout questionnaire
SOFI: Swedish occupational fatigue inventory
UFC: 24 h Urinary free cortisol
