# Erratum to: Adrenal fatigue does not exist: a systematic review

**DOI:** 10.1186/s12902-016-0132-8

**Published:** 2016-11-16

**Authors:** Flavio A. Cadegiani, Claudio E. Kater

**Affiliations:** Adrenal and Hypertension Unit, Division of Endocrinology and Metabolism, Department of Medicine, Escola Paulista de Medicina, Universidade Federal de São Paulo (EPM/UNIFESP), R. Pedro de Toledo 781– 13th floor, 04039-032 São Paulo, SP Brazil

## Erratum

Following publication of the original version of this article in BMC Endocrine Disorders [1] it was brought to our attention that the comment section for row 12 in Table 1 should be blank. Please find the corrected table below.

**Table 1:** Summary of selected studies

First author & reference	Year of publication	Number of patients	Population	Questionnaire	Tests	Results	Comments
McLennan [21]	2016	257	H/B	Maslach	HCC	Nl	
Schmidt [69] (part 1)	2016	265	Breast Cancer	FAQ	DAC, CAR, SCR, NSC	↑NSC, ↑AUC, Nl CAR, Nl DAC, ↓SCR	Pts. mostly on chemotherapy (ChTx); schemes not specified. No controls, only correlation between fatigue levels and tests. ChTx related to worse fatigue. Initial tests performed during, w/ or w/o ChTx.
Schmidt [69] (part 2)	2016	265	Breast Cancer	FAQ	DAC, CAR, SCR, NSC	↑AUC, ↑CAR, ↑NSC, ↑ SCR, Nl DAC	Second test of the study, performed after 14 weeks of procedures.
Sjors [25]	2015	220	H/B	SMBQ	DAC, CAR	↓CAR, ↑DAC	Results normalized after adjustment of anti-depressive use; SCR results not provided
Oosterholt [28]	2015	91	H/B	Maslach	DAC, CAR, SCR, AUC, CAR 60min	Nl SCR, ↓CAR, Nl AUC, Nl CAR 60min, ↓DAC	Control of variations did not change results
De Vente [22]	2015	95	H/B	Acute Psychosocial Stressor diagnosis	MSC, MST	Nl MSC, Nl MST	MST: Nl (women), reduced (men); MSC: Nl (men), reduced (women). Mental arithmetic and public speech stressors also performed
Lennartsson [24]	2015	56	H/B	DCSRD; exclusion w/ SEQ	DHEA-S; ACTH; ACTH and DHEA-S post-TSST (TSST stress test performed)	↓DHEA-S, ↓post-TSST DHEA-S, Nl ACTH, Nl post-TSST ACTH	
Tao [23]	2015	171	H/B	Maslach	MSC, ACTH	↑ACTH, ↑MSC	
Jonsson [29]	2015	51	H/B	SMBQ	MSC, MST	Nl MSC, ↓MST	
Lennartsson [27]	2015	56	H/B	DCSRD and SMBQ	MSC, MST, ACTH, ACTH post-TSST (TSST stress test performed)	Nl MSC, Nl MST, Nl ACTH, Nl post-TSST ACTH	Severe BO: lower ACTH, cortisol response to TSST vs controls, whereas low BO: higher ACTH, cortisol responses vs controls
Schmaling [26]	2015	62	Healthy	ADAS	AUC, SCR	↓AUC, ↓SCR	31 couples studied, one of which had chronic fatigue (but not CFS)
Sveinsdottir [68]	2015	1150	Chronic Lombalgia	CFQ	DAC, CAR, SCR, NSC	Nl DAC, Nl CAR, Nl NSC, Nl SCR	
Powell [67]	2015	76	Multiple Sclerosis	CFQ	DAC, CAR, SCR	↓DAC, ↑CAR, Nl SCR	Sleep disorders excluded; adjusted for depressive symptoms; NSC not published. Multiple sclerosis had increased awakening cortisol and decreased CAR
Cruz [70]	2015	43	Breast cancer	BFI and CFQ	MSC, DHEA-S	Nl MSC, Nl DHEA-S	Pts undergoing ChTx included anthracyclines
Marchand [31]	2014	1043	H/B	Maslach	DAC, CAR, SCR, NSC	↑DAC, ↓CAR, ↑SCR, ↓NSC	Only BO; Groups of severe distress or depression not included
Aggarwal [30]	2014	227	Healthy	CFQ	MSC, NSC, 0.25mg DST, DHEA-S	Nl MSC, Nl NSC, Nl 0.25mg DST, ↓DHEA-S	Evaluation of chronic, widespread pain, chronic orofacial pain, chronic fatigue (but not CFS), irritable bowel syndrome
Tell D [71]	2014	130	Breast cancer	MFI	DAC, CAR, SCR, NSC	↓CAR, ↓SCR, ↑DAC, ↑NSC	Post-surgery breast cancer, regardless of ChTx. Not adjusted to sleeping patterns
Wolfram [32]	2013	53	H/B	Maslach	MSC, 1mcg CST, DST/CRH test (1.5mg DST + 100mcg CRH)	Nl MSC, ↓post-1mcg ACTH, Nl ACTH and cortisol DST-CRH	High over-commitment present blunted serum and salivary cortisol and ACTH responses to DST-CRH test
Klaassen [33]	2013	27	Healthy	MP (Beck & Luine, 2010) and Stress Tasks (Wang, 2006)	MSC, post-stress	Nl MSC, Nl post-stress	A complex test sequence was performed but not reproduced
Eek [34]	2012	581	Healthy	SOFI-20	DAC, CAR, NSC, SCR, MSC	Nl DAC, Nl CAR, Nl NSC, Nl SCR, Nl MSC	Women: reduced awakening, increased CAR, increased SCR; Men: increased awakening and reduced CAR – when fatigued
Sjors [35]	2012	247	H/B	DCSRD and SMBQ	DAC, 15min CAR	Nl DAC, Nl 15min CAR	
Rahman [54]	2011	30	CFS	Previous Dx – No questionnaire	MSC, SCR, NSC	Nl MSC, Nl SCR, Nl NSC	
Moya-Albiol [37]	2010	64	H/B	Maslach	DAC, CAR	Nl DAC, Nl CAR	
Kumari [38]	2009	4,299	Healthy	SF-36	DAC, CAR, NSC, SCR	↓DAC, ↓CAR, ↑NSC, ↓SCR	Adjusted for WC*, BMI, sleep duration, CVD medication, depressive symptoms, smoking, alcohol intake provides Nl awakening but lower SCR
Osterberg [39]	2009	304	H/B	Maslach	DAC, CAR, NSC, SCR	Nl DAC, Nl CAR, ↓NSC, ↑SCR	0.5mg DST was not compared to controls
Wingenfeld [41]	2009	279	H/B	Maslach and Maastricht	AUC, SCR	Nl AUC, Nl SCR	DAC and CAR not done; conclusions different from results. For AUC, Low BO: Nl, moderate: increased, severe: decreased
Rydstedt [40]	2009	76	Healthy	NRWS	DAC, NSC	Nl DAC, Nl NSC	
Papadopoulos [55]	2009	38	CFS	CFQ and SF-36	MSC, AUC, morning AUC, 0,5mg DST	↑MSC, ↑AUC, ↑MAUC, Nl 0.5mg DST	Data on absolute cortisol levels at each point not published. DST reduction evaluated by percent reduction.
Bay [72]	2009	75	Post traumatic brain injury	POMS	AUC	Nl AUC	Correlation between brain injury-related fatigue level and cortisol AUC. Basal and NSC results not reported; SCR not evaluated.
Sudhaus [73]	2009	43	Chronic Lombalgia	MFI	DAC, CAR, MAUC	↓CAR, Nl DAC, Nl MAUC (correlation between fatigue levels among low back pain subjects)	** Colocar como SAUDÁVEL – porque é só lombalgia (Low back pain had increased CAR than controls).
Lindeberg [36]	2008	78	Healthy	SF-36	DAC, CAR, NSC, SCR	Nl DAC, ↓CAR, Nl NSC, ↓SCR	
Sertoz [42]	2008	72	H/B	Maslach	Basal and post 1.0mcg DST cortisol	Nl basal cortisol and 1.0mg DST	
Bellingrath [43]	2008	101	H/B	Maslach and Maastricht	DAC, CAR, NSC, SCR, 0.25mg DST	Nl DAC, Nl CAR, Nl NSC, Nl SCR, ↓0.25mg DST	
Nater [57]	2008	185	CFS	SF-36 and MFI	DAC, CAR, MAUC	Nl DAC, Nl CAR, ↓MAUC	
Torres-Harding [56]	2008	108	CFS	FSE	AUC, SCR	Nl AUC, Nl SCR	Multiple psychological tests performed. Data on NSC, basal and CAR not published.
Sonnenschein [45]	2007	42	H/B	Maslach	CAR, 0.5mg DST, DHEA-S	Nl CAR, Nl 0.5mg DST, Nl DHEA-S	Adjusted for depression, sleep quality. Awakening levels and each level graphics not available
Harris [44]	2007	44	Healthy	SF-36	DAC, CAR, NSC, SCR	Nl DAC, Nl CAR, Nl NSC, Nl SCR	Other aspects also correlated: complains, job stress and demand, QOL and coping. Adjusted for coffee and tobacco.
Langelaan [46]	2006	55	H/B	Maslach	DAC, CAR, 0.5mg DST, DHEA-S	Nl DAC, Nl CAR, Nl 0.5mg DST, Nl DHEA-S	Engaged work also compared and had stronger suppression in DST
Mommersteeg [47]	2006	109	Healthy	NC-WHO	DAC, CAR, 0.5mg DST, SCR, AUC, NSC	Nl DAC, Nl CAR, Nl NSC, Nl SCR, Nl 0.5mg DST, Nl AUC	
Barroso [74]	2006	40	HIV	HRFS	MSC, NSC	↓MSC, ↑NSC	
Jerjes [58]	2006	80	CFS	CFQ	UFC, TCM	↓UFC, Nl TCM	
Grossi [48]	2005	64	H/B	SMBQ	DAC, CAR	↓DAC, ↑CAR	Groups were high x moderate x low BO score; correlation was significant
Segal [59]	2005	40	CFS	No questionnaire	MSC, 1mcg CST	↓ MSC, ↓1mcg CST	DHEA-S collected only in CFS. No questionnaires used.
Jerjes [60]	2005	35	CFS	CFQ	MSC, SCR, NSC, AUC	↓MSC, ↓SCR, ↓AUC ↓, Nl NSC	
Bower [75]	2005	29	Breast cancer	SF-36	DAC, AUC, SCR, NSC	↑AUC, ↓SCR, Nl DAC, ↑NSC	Post-ChTx (regardless of time) complete cancer remission and exclusion of other disorders
McLean [76]	2005	55	Fybro-mialgia	SF-36	DAC, 60min CAR, SCR, AUC, NSC	Nl DAC, Nl 60min CAR, Nl SCR, Nl AUC, Nl NSC (correlation between fatigue levels among FMG subjects)	FMG subjects presented Nl DAC and CAR, as controls.
Roberts [62]	2004	92	CFS	CFQ and SF-36	DAC, CAR, MAUC	Nl DAC, ↓CAR, ↓MAUC	
Crofford [61]	2004	72	CFS/ FMG	POMS	ACTH, MSC, SCR, NSC, AUC	Nl ACTH, Nl SCR, Nl NSC, ↓AUC, Nl MSC	Tests performed in: CFS, FMG and CFS + FMG; FMG w/o fatigue had Nl AUC and increased BMC levels
Moch [50]	2003	16	H/B	Maslach	UFC, DHEA-S, ACTH, MSC	↓UFC, Nl DHEA-S, Nl ACTH, ↓MSC	Only women; longitudinal evaluation – Nl initial cortisol.
De Vente [49]	2003	45	H/B	Maslach	DAC, MSC, post-TSST	↑DAC, ↑MSC, Nl post-TSST	
Gaab [63]	2002	42	CFS	MFI	DAC, CAR, SCR, 0.5mg DST	↓0.5mg DST, Nl AUC, Nl CAR, Nl DAC, Nl SCR, Nl NSC	CAR also performed at 15, 45 and 60min.
Dekkers [77]	2000	53	Rheumatoid Arthritis	MFI	DAC, CAR, SCR, AUC	Nl AUC, Nl SCR Nl, ↓DAC, ↑CAR	5/25 subjects with RA taking prednisone (5–10 mg/d); RS subjects had smaller SCR, increased AM cortisol and decreased CAR. 15 and 45 min CAR also performed.
Melamed [51]	1999	111	H/B	SMBQ and Maastricht	MSC and 4PM cortisol	↑MSC, Nl 4PM cortisol	
Pruessner [52]	1999	66	Healthy	Maslach	DAC, CAR, 0.5mg DST	↓DAC, ↓CAR, ↓0.5mg DST	15min and 60min CAR also performed
Strickland [65]	1998	74	CFS	Not specified/detailed	MSC, NSC	↓NSC, Nl MSC	Adjusted for depression
Young [66]	1998	45	CFS	NC-WHO	UFC, SCR, MSC, AUC	Nl UFC, Nl SCR, Nl MSC, Nl AUC	
Scott [64]	1998	28	CFS	Not specified (not detailed)	MSC, ACTH, 100mcg CRH cortisol stimulation	Nl MSC, Nl ACTH, CRH stim test: ↓cortisol, ↓ACTH	
Raikkonen [53]	1996	22	Healthy	Not assessed	1mcg CST, DST (non specified), OGTT, MSC, ACTH, cortisol/ACTH ratio	↑Cortisol/ACTH ratio; ↑CST, Nl DST, Nl OGTT, Nl MSC, Nl ACTH	Full article not assessed – not in PUBMED or other database

Questionnaires: *SMBQ* Shirom-Melamed Burnout Questionnaire, *BFI* Brief Fatigue Inventory, *CFQ* Chalder Fatigue Questionnaire, *Maslach* Maslach Burnout Inventory, *SF-36* Short Form Health Survey 36, *NC-WHO* Neurasthenia Criteria, *DCSRD* Diagnosis Criteria of Stress-related Exhaustion Disorder, *SEQ* Stress-Energy Questionnaire, *ADAS* Abbreviated Dyadic Adjustment Scale, *MFI* Multidimensional Fatigue Inventory, *FAQ* Fatigue Assessment Questionnaire, *MP* Memory Performance, *POMS* Profile of Mood States, *Stress Tasks*; *FSE* Fatigue Severity Scale, *SOFI* Swedish Occupational Fatigue Inventory, *Maastricht* Maastricht Vital Exhaustion Questionnaire, *NRWS* Need for Recovery from Work Scale, *HRFS* HIV-Related Fatigue Scale

Other abbreviations: *CFS* Chronic Fatigue Syndrome, *H/B* Healthy/Burnout, *24h-UFC* 24-h Urinary Free Cortisol, *FMG* Fibromyalgia, ↑: Increased or elevated, ↓: Decreased or reduced, →: Unchanged, *Nl* Normal
